# A qualitative exploration of further education college staff perspectives on the challenges and opportunities for using physical activity to support student mental health in England

**DOI:** 10.1186/s12982-026-02817-x

**Published:** 2026-09-06

**Authors:** Andy Mahon, Ciara Thomas, Russell Jago, Judi Kidger, Laura Tinner

**Affiliations:** 1 Centre for Public Health, University of Bristol, Canynge Hall, Clifton, Bristol, BS8 2PL UK; 2 NIHR Bristol Biomedical Research Centre, University Hospitals Bristol and Weston NHS Foundation Trust and University of Bristol, Bristol, UK

**Keywords:** Physical activity, Mental health, Young people, Further education

## Abstract

**Background:**

The increasing prevalence of poor mental health amongst young people is a public health concern, and physical activity (PA) is a potential intervention that has both physical and mental health benefits. While education settings are important contexts for intervention delivery, little is known about the role of PA in student mental health in Further Education (FE) Colleges. This qualitative study aimed to explore the perspectives of college staff regarding the role of PA to support student mental health.

**Methods:**

Twenty nine college staff from 5 colleges in the South-West of England took part in semi-structured interviews. Interviews were recorded, transcribed and anonymised, and then analysed using the Framework Method.

**Results:**

Staff perceived barriers to using PA to support student mental health in FE colleges were identified across four key themes; a missing link between PA and mental health, physical activity having no core place in further education, negative attitudes towards physical activity and practical limitations to physical activity provision. Opportunities identified for future implementation included a more joined-up approach between PA and mental health, systemic changes to policy and funding structures, and strategies to address student-level barriers to improve engagement with PA at college.

**Conclusions:**

While PA is a potential preventative and supportive mental health strategy within FE colleges, staff suggested meaningful future implementation requires structural, cultural and policy-level changes to ensure equitable access and engagement. Future research should explore young people’s perspectives and evaluate recommended strategies for embedding PA within FE to support college student mental health.

**Supplementary Information:**

The online version contains supplementary material available at https://doi.org/10.1186/s12982-026-02817-x.

## Introduction

Mental health challenges amongst young people are a growing public health problem. Globally, mental health disorders are now among the leading causes of ill-health and disability in adolescents and young adults [1, 2]. Rates of poor mental health in the UK have risen substantially, with recent figures estimating that one in five young people aged 16 to 24 experience a diagnosable mental health condition [3]. Mental health difficulties at this life stage can have profound and long-lasting impacts on personal and social development and educational and employment outcomes [4]. Given the increasing prevalence of problems and limited capacity of existing mental health services, there is an urgent need to identify alternative accessible and scalable approaches outside of the healthcare system to support the mental health of young people.

Physical activity, defined as “any bodily movement produced by skeletal muscles that results in energy expenditure” [5], is a promising mental health intervention option for young people. Regular physical activity is associated with mental health-related benefits in adolescents and young adults, such as improvements in stress, depression, anxiety and overall wellbeing, increased self-esteem and improved mood [6, 7]. However, many young people may not be sufficiently active to achieve these benefits as almost one in three FE students in England are not engaged in enough physical activity to meet recommended guideline amounts [8]. The evidence suggests physical activity-based interventions delivered within education settings could be feasible and effective for supporting a wide range of mental health-related outcomes, offering a potential reach to large numbers of young people in a familiar environment [9, 10]. Prior work has shown physical activity can support student mental health in two complimentary ways: (1) as a universal mental health promotion strategy, to promote and maintain good mental health, and (2) as a targeted or supportive intervention option for students already experiencing mental health difficulties [9]. However, there is a lack of previous research exploring how physical activity could be utilised within the Further Education (FE) sector in England as a mental health promotion or treatment strategy.

FE refers to the post-16 education stage between secondary school and higher education and employment in England, corresponding to the OECD classification of upper secondary education (ISCED level 3) [11]. Students can choose a wide range of academic, vocational or technical study options, in several different settings, including FE colleges or school 6th forms. FE colleges cater for around 3 million students in England, and are often characterised by high student diversity, including a high proportion of neurodivergent students, students with special educational needs and/or learning disabilities and many students from diverse socioeconomic backgrounds [12, 13]. A recent report showed 95% of English FE colleges reported a growing prevalence of mental health problems amongst their students [14]. However, little is currently known about how FE staff understand the role of physical activity in supporting student mental health, or how they perceive the feasibility, barriers and opportunities for using physical activity in this context. Given this increasing prevalence of mental health difficulties amongst FE students, the established mental health benefits of physical activity and the paucity of research examining how physical activity may be used to support student mental health within college settings, we conducted this study to inform future practice, policy and intervention development.

This study explored the perspectives of FE college staff on the role of physical activity in supporting student mental health, and challenges and opportunities in this area. Although our focus is on the FE context, young people’s engagement in physical activity is shaped by a wider set of individual, social, and environmental factors beyond the college setting, awareness of which will help inform future work within FE settings [15]. Specifically, the objectives were to:

- Identify College staff’s perspectives on the barriers to the provision and/or implementation of physical activity initiatives to support student mental health.
- Explore College staff’s insights about opportunities that may exist to support the mental health of college students through physical activity in the future.

## Methods

### Research design

A qualitative design was chosen due to the exploratory nature of the study aims, enabling detailed insights into staff experiences and views. This approach provided flexibility in exploring the study’s aims, whilst also giving participants the freedom to raise important views that may have not been encompassed by the study objectives.

An interpretivist orientation underpinned the research, recognising that participants’ perspectives on physical activity and student mental health are shaped by their roles, institutional contexts and lived experiences within FE settings. This orientation aligned with our aim of understanding how staff interpret and make sense of physical activity and mental health within college contexts.

### Participant recruitment

#### Sampling and sample size

To be eligible to participate, participants must, firstly, have been current employees at a College of Further Education in the South-West of England at the time of recruitment, between May and August 2024. Secondly, they must have been employed in a role where they worked directly with students or a role directly related to physical activity or mental health service provision or management, to ensure they possessed the relevant knowledge to meet the study aims. Purposive sampling was conducted to ensure participants met these eligibility criteria, had the necessary knowledge and insight to meet the aims of the study, and to ensure a diverse range of insights from staff with different levels of experience, roles and gender and from different FE colleges. Snowball sampling was also utilised as recruitment and data collection progressed, based on participants’ recommendations for colleagues with valuable insights, or different roles. The adequacy of the sample size was guided by the concept of information power, considering the broad aims of the study, sample characteristics, quality of dialogue and the planned analytical strategy [16]. As data collection progressed, the breadth of roles represented within FE and the complexity of the topics warranted the inclusion of a diverse range of perspectives.

Emerging findings further highlighted the importance of recruiting information-rich participants, particularly those in senior leadership roles which was supported through snowball sampling. Recruitment continued until sufficient information power was judged to have been achieved, when the interviews had provided rich and relevant insights across a diverse range of staff roles, and the breadth and depth of perspectives were sufficient to address the study aims.

#### Recruitment setting and procedure

Participants were recruited from five college campuses in the South-West of England. The participating colleges were located across both rural and urban contexts and were a mix of large and medium-sized general colleges of further education and sixth-form colleges. While approximately 3500 staff members were employed across the five colleges, we were unable to determine the specific population size from which the sample is drawn because not all employees were eligible if they were not in student facing roles related to physical activity and mental health.

Each participating college provided site approval, with a senior member of staff acting as a gatekeeper for the study. These key contacts collaborated with the research team to approve and facilitate recruitment by distributing the study information and recruitment materials, including advertisements, digital posters and an information video, to staff at their college by email. The participant information sheet, shared during recruitment, explained that a £25 digital voucher would be provided as a token of appreciation for taking part following the interview. Interested staff contacted the research team directly for more information via email or phone, or registered to take part via an online consent form. Those who registered were asked to complete a short online demographic survey, which recorded relevant details to inform purposive sampling, including their length of time working at the college, title of their role, gender and the name of the college they were employed at. Based on these data, eligible participants were selected to ensure diversity across roles, experience, and college contexts, which was reviewed and discussed as a research team prior to selecting each subsequent participant. Selected participants were contacted by their preferred means (email or telephone) to arrange their interview.

#### Participants

Of the 39 individuals who expressed interest, six individuals did not proceed due to non-response or withdrawal (four did not respond to repeated attempts to schedule an interview, while two did not attend their scheduled interviews and did not respond to follow-up contact). A further four individuals were not selected, as they possessed characteristics already well represented within the emerging sample, including type of role and college of employment, and were therefore not selected to maintain diversity across roles, experience, gender, and college contexts. Therefore, a total of 29 participants took part in interviews. Following the review of one interview, it was decided to remove that participant’s data from the analysis due to concerns about maintaining anonymity and confidentiality. Therefore, the final sample consisted of 28 participants. Table 2 provides an overview of participant characteristics.

### Data collection

Data were collected through interviews between May and August 2024 either online (*n* = 28) or in-person (*n* = 1). Interviews were conducted in a semi-structured format, guided by a topic guide (Supplementary material 1) which was developed for the study by the research team, and informed by relevant literature and the aims and objectives of the study. Additional input was obtained in consultation with a local authority public health consultant and a college staff member (who was not eligible to participate due to location). Key contacts at each participating college provided contextual insights about local priorities and student mental health which also informed development of the topic guide, however, this limited input did not make them ineligible from subsequently taking part in the study. The topic guide asked about staff’s perspectives and experiences of student engagement with physical activity, student mental health, current physical activity and mental health initiatives within colleges, and opportunities for using physical activity to support student mental health in the future. The topic guide was used flexibly, with the sequencing adapted to participant’s professional roles to ensure questions were relevant and meaningful. For example, interviews with staff in mental health support roles began with mental health-focused questions, whereas those working in sport or physical activity began with questions relating to physical activity. All core topics were explored in each interview. Each interview lasted between 45 and 60 minutes. After the interviews, participants were sent a thank you email containing a £25 digital shopping voucher.

The interviews were conducted by the first author (AM), a PhD student who, although had limited prior interview experience, received training in qualitative interviewing methods as part of doctoral research training and ongoing supervision from experienced qualitative researchers from the research team. The interviewer had no prior relationship with participating colleges, and prior to participating, individuals received the participant information sheet outlining the study aims, the role of the researcher and AM’s status as a PhD researcher investigating physical activity and mental health within FE settings. AM has academic and professional experience in mental health and wellbeing promotion, a personal interest in physical activity and sport, and did not attend college in England. These experiences will have influenced assumptions regarding the value of physical activity for mental health and shaped interpretations. A reflexive approach was adopted throughout, including keeping a reflexive journal, and research team discussions about how our backgrounds and assumptions inform decisions and interpretation. To support reflexive interpretation and minimise the influence of any single researcher’s perspective, the coding framework was developed through an iterative process informed by the independent coding of three transcripts by two researchers with no physical activity background (LT & CT), with subsequent regular team discussions to explore different interpretations and refine themes as data analysis progressed. This approach strengthened the credibility and depth of the analysis. Credibility was also supported through member checking whereby participants were sent a summary of the preliminary findings and invited to comment on the interpretations; several participants responded, confirming that the findings reflected their experiences.

### Data analysis

Data were analysed using the Framework Method, a structured form of thematic analysis [17]. This method was chosen because it aligned well with the applied focus of the study and the need to compare perspectives across a diverse sample of staff working in different roles and FE college contexts. Its structured yet flexible approach accommodates both inductive and deductive coding, supporting the exploration of emergent ideas while addressing the study objectives. The matrix-based structure provided a transparent analytic process and facilitated a collaborative, team-based approach, strengthening the rigour and coherence of the analysis. This method allows for an inductive-deductive approach, with coding and analysis informed by both the study aims and by concepts interpreted directly from participants’ accounts [17]. The seven step process used for the Framework Method for analysis is outlined in Table 1 [17].

**Table 1:** Overview of the 7-steps followed as part of the framework approach to analysing the data

Step	Description
1. Transcription:	Each online interview was digitally transcribed using Microsoft Teams’ transcription feature. The in-person interview (*n* = 1) was recorded using a University-approved recording device, and transcribed verbatim by the lead researcher. One researcher (AM) manually corrected and anonymised each transcript by comparing the video/audio recording and transcript line-by-line, correcting any transcription errors and removing any identifiable information.
2. Familiarisation:	AM became familiar with each interview by re-watching each video recording. Any relevant observations or thoughts at this point, such as participant body language or non-verbal communications, such as facial expressions, gestures, pauses or emphasis on specific words, and important views or points they shared were noted, using both the comments feature on the digital file, and separately in an analysis logbook document. Once fully corrected and anonymised, each transcript was then read and re-read fully to ensure familiarisation.
3. Coding:	All transcripts were uploaded to Nvivo14 software for coding. The analysis followed an inductive-deductive approach with codes developed both based on the study aims and objectives and emerging from participant’s accounts within the data. An iterative coding process was followed, whereby initial codes were generated through close reading of a subset of transcripts. For this, one researcher (AM) initially line-by-line coded five transcripts to develop an initial coding framework.
4. Developing a working framework:	Once this initial coding framework was developed, three researchers independently coded a further three transcripts (AM, LT, CT) using the initial coding framework. The independently coded transcripts were then compared in detail, and areas of overlap, disagreement, and ambiguity were discussed collaboratively. These discussions enabled the refinement of code definitions, the merging of repeated and overlapping codes and the identification of new codes where needed, to enhance credibility and ensure that it captured both anticipated and emergent concepts. The involvement of multiple researchers at this stage was used as a reflexive resource to explore different interpretations and challenge individual assumptions, rather than to seek agreement in a quantitative sense. This process enhanced analytic rigour by integrating multiple perspectives and reducing the influence of any single researcher’s assumptions.
5. Applying the analytic framework:	This refined coding framework was then systematically applied to the remaining transcripts, with line-by-line coding completed by the lead researcher (AM). The process remained iterative throughout: as coding progressed, interpretations, insights, ambiguities, or new nuances were discussed with the wider research team. These discussions informed further refinement and reorganisation of codes where necessary. This reflective and iterative process continued throughout, progressing until the final transcript had been fully coded. Any adjustments to the framework were applied consistently across previously coded transcripts to maintain analytic coherence.
6. Charting the data into the framework matrix:	Once fully coded, the data were charted into a framework matrix in NVivo14. The software creates a matrix of cases (transcripts) as rows and codes as columns, with each cell containing the text coded to that specific code. The data within each cell was summarised into concise analytic summaries that captured key points while retaining the nuance and meaningful insights of the original text. Column summaries were then produced to synthesise the insights within each code across all participants; these were used to identify overarching patterns and informed the development of higher‑level themes in the subsequent interpretive stage. Charting enabled systematic cross‑case comparison across staff roles, backgrounds, and college contexts.
7. Interpreting the data:	The matrix was used to compare and contrast coded data across participants, roles and college contexts to identify patterns, similarities and differences. This was facilitated by running matrix queries in NVivo14. Column summaries were examined to explore how related codes clustered together and to interpret the underlying concepts represented. Visualisations in the forms of spider graphs (both digital and handwritten) supported the exploration of connections between codes and patterns. Provisional themes were developed from these concepts and refined through team discussions to ensure they represented coherent and meaningful insights across the dataset. Participant feedback on preliminary findings further supported the coherence and credibility of the developed themes.

### Ethical considerations

This study received ethical approval from the University of Bristol Faculty of Health Sciences Research Ethics Committee (FREC), study reference number 17747 and was conducted in accordance with the principles of the Declaration of Helsinki. Each college provided written approval for their staff to participate in the study. All participants provided written informed consent before the interview commenced. To ensure confidentiality and anonymity for participants, all transcripts were anonymised prior to analysis.

## Results

A total of 28 participants were included in the analysis, details of which are presented in Table 2 below.

**Table 2:** Participant characteristics and representation across colleges

		Total	College 1	College 2	College 3	College 4	College 5
Total *N*:		28	6	8	3	6	5
**Gender**:							
Male		7	1	2	-	-	4
Female		21	5	6	3	6	1
**Experience – length of time in current role within the college**:							
0–1 years		3	1	-	-	1	1
2 to 5 years		11	3	3	1	2	2
5 to 10 years		4	-	2	1	-	1
10 years or more		10	2	3	1	3	1
**Participants role within college**:							
Mental health & wellbeing / pastoral care staff		7	1	1	1	4	-
Teacher/lecturer		8	2	3	1	1	1
Course/curriculum area manager		5	1	2	-	-	2
Director(s)/Department Head/Senior Leadership Team Member		7	2	2	1	1	1
Sports coaching staff		1	-	-	-	-	1

Table 2: *overview of participant demographic details and college representation. Colleges are anonymised as Colleges 1–5 to protect institutional identity. Role categories are intentionally broad to preserve participant confidentiality.*

From the interviews, we interpreted four themes reflecting participants’ perspectives on barriers and opportunities to physical activity as a mental health support strategy for college students: A missing link between physical activity and mental health, physical activity having no place in further education, negative attitudes towards physical activity and practical challenges to physical activity provision. These are described in the following sections, illustrated by participant quotations. Table 3 provides anonymised participant identifiers and key characteristics used when presenting quotations in the Results section.

**Table 3:** Anonymised participant identifiers and key characteristics used when presenting quotes in the results section

Participant	Role category	Experience – length of time in current role within the college	Gender
P01	Course/curriculum manager	2 to 5 years	Male
P02	Teacher/Lecturer	10 years or more	Female
P03	Teacher/Lecturer	0 to 1 years	Female
P04	Director(s)/Department Head/Senior Leadership Team Member	2 to 5 years	Female
P05	Teacher/Lecturer	2 to 5 years	Female
P06	Mental health & wellbeing / pastoral care staff	2 to 5 years	Female
P07	Mental health & wellbeing / pastoral care staff	10 years or more	Female
P08	Mental health & wellbeing / pastoral care staff	0 to 1 years	Female
P09	Course/curriculum manager	5 to 10 years	Female
P10	Mental health & wellbeing / pastoral care staff	0 to 1 years	Female
P11	Mental health & wellbeing / pastoral care staff	2 to 5 years	Female
P12	Teacher/Lecturer	10 years or more	Female
P13	Mental health & wellbeing / pastoral care staff	10 years or more	Female
P14	Course/curriculum manager	2 to 5 years	Male
P15	sports coaching staff	10 years or more	Male
P16	Mental health & wellbeing / pastoral care staff	10 years or more	Female
P17	Teacher/Lecturer	10 years or more	Female
P18	Course/curriculum manager	10 years or more	Female
P19	Teacher/Lecturer	2 to 5 years	Female
P20	Director(s)/Department Head/Senior Leadership Team Member	5 to 10 years	Male
P21	Director(s)/Department Head/Senior Leadership Team Member	5 to 10 years	Male
P22	Director(s)/Department Head/Senior Leadership Team Member	2 to 5 years	Female
P23	Teacher/Lecturer	10 years or more	Female
P24	Director(s)/Department Head/Senior Leadership Team Member	10 years or more	Male
P25	Director(s)/Department Head/Senior Leadership Team Member	2 to 5 years	Male
P26	Teacher/Lecturer	2 to 5 years	Female
P27	Director(s)/Department Head/Senior Leadership Team Member	2 to 5 years	Female
P28	Course/curriculum manager	2 to 5 years	Female

### Theme 1: “a missing link” between physical activity and mental health

Staff described a ‘missing link’ between physical activity and mental health in Further Education, noting that the two were rarely connected in students’ thinking, within college structures and practices or across the sector.

### Student-level disconnect between physical activity and mental health

Participants felt that physical activity and mental health are rarely conceptualised as connected issues for students. Staff perceived that students who regularly engaged in physical activity were less likely to seek support for mental health concerns. However, they suggested that these impacts were much less evident to students themselves, that they *‘can’t see it that the ones who do things (physical activities) are the ones who are better off’ [018]*, noting that students typically view physical activity and mental health as separate

*I think when you talk about physical activity*,* students just think of it as physical. I don’t think they understand the impact on their mental health. [P08]*

For some, this went deeper than a lack of awareness or education. Rather, there is a perceived lack of belief in the potential benefits, and a sense that many students do not think physical activity *‘would work for them’ [P12]*. Several staff members discussed how, at a basic level, most people have been told that ‘exercise is good for you’, but amongst students, this knowledge is often not translated into action. As a result, students typically do not recognise physical activity as a potential means for supporting their mental health or wellbeing.

Participants suggested a key practical strategy for the future was to promote more clearly that physical activity and mental health are two interlinked components of health:

*The biggest thing is making them aware of the good and the positive impacts that sort of physical activity can have on your mental health [P08]*.

Staff proposed that an educational component could help connect these elements in students’ thinking. The induction when a student arrives for their first year of college was recognised as a good starting point; providing an immediate education and awareness to new students, highlighting that physical activity and mental health are linked as part of holistic health:

*So it’s kind of making it more explicit*,* that’s promoted at the start…in the induction*,* literally like in the first two weeks. This is how we work…we are interested in student mental health. [P26]*

As all students are paired with a tutor and attend tutorials as part of their pastoral care and personal development through their college journey, this could be an opportunity to insert this awareness building into a pre-existing structure already within students’ timetables. Staff also recommended more regular and widespread promotion of physical activity opportunities alongside this throughout the college year.

### College‑level disconnect between physical activity and mental health

Participants further observed that physical activity and mental health were structurally disconnected within colleges, rarely thought of together, and seen as a missed step in developing more joined‑up approaches to student wellbeing. While physical activity and mental health were seen as siloed at a structural level within colleges, participants suggested building partnerships between these departments and developing shared working plans and responsibilities. Through increased collaboration and shared ownership, staff felt colleges could support students in a more holistic way. Participants proposed physical activity could also be formally integrated into the student support model, including wellbeing teams that signpost and refer students presenting for mental health support to sport or activity opportunities and coaches. However, staff noted that physical activity may not be an attractive option for all students, and that the college needs to respect students’ choices. Staff were also cautious to highlight that while physical activity could be appropriate for many students presenting with low level concerns, there would still need to be explicit mental health support in place for those with more moderate-to-severe needs.

Overall, staff highlighted that these two elements need to be better connected at the college level, from those in senior leadership positions downwards, to enable students’ development to be viewed in a more holistic way.

### Theme 2: a perception that physical activity is not a core part of further education

#### The peripheral role of physical activity in further education

Participants identified an issue around physical activity having no central place in FE, which contributed to the above lack of connection and integration between physical activity and mental health. It was felt that physical activity is not prioritised, and rather it is an optional extra there only for those who are passionate about sports:

*Unfortunately physical activity*,* sport*,* yoga*,* dance…Just movement has traditionally maybe not been an idea in FE. [P28]*

*No*,* it’s (physical activity) not been a priority. It comes back to what keeps you in business and that for us is bums on seats and grades. [P14].*

These comments suggested a unique and implicitly understood physical activity ‘culture’ within the FE sector and colleges, which shapes attitudes and behaviours of the staff and students.

*It’s (physical activity) not really a part of the culture of college in any way…I’m not aware of much that goes on in the college to promote that whatsoever. We all know how good it is for mental health to be active*,* and I don’t think there’s any encouragement to do that whatsoever. [P17]*

Here, this *‘cultural legacy’ [P05*] refers not to an environment that actively values and encourages physical activity, but to a set of norms within further education that places physical activity as outside the core priorities of the sector. This traditional sense that physical activity was not a valued, core part of FE has positioned it as an optional and non-essential component in comparison to academic outcomes. Specifically, high workloads and additional revision alongside busy timetables were discussed as making it difficult to engage with physical activities during college time. This is compounded by the lack of timetabled opportunities, in comparison to schools in England which have scheduled compulsory physical education. An example of this physical activity culture of FE includes the use of mandatory enrichment hours, which are a primary physical activity opportunity for most students but participants noted that these timetabled hours were being encroached upon by scheduling further revision and classes. This was seen as particularly relevant to students on more academic courses, where revision and work was heavily prioritised over “extras” [P28] like physical activity:

*There’s revision. There’s extra work that can be done*,* there’s studying that can be done…let’s not bother with enrichment*,* what’s the point? I know there are sports people around the college that cannot attend because they have to study [P15].*

Even students who regularly take part in physical activities tend to drop them when workloads are high. This was discussed as a key issue, as these exam periods are often highly stressful and participants felt dropping these healthy routines and pressure ‘*outlets’ [P18*] often compounded the stress for students. Staff also mentioned this was often a result of external pressure:

*I also think parents have a massive effect… parents only care about their grades. They can’t possibly play sports as well” [P15].*

One key feature of FE colleges is that they may offer specialist courses not delivered elsewhere and as such students often travel long distances to attend the course, with long commutes eating into personal time. Many also try to balance part-time jobs alongside their studies or have driving lessons as they enter late adolescence to gain independence and support their families:

*A lot of them will have part time jobs …money is obviously quite important to a lot of them… so actually*,* they’re straight off campus*,* off to work. [P07]*

Staff called for stronger protection of enrichment hours for all students, suggesting that directives should come from the college leaders to ensure these timetabled slots are ‘off-limits’:

*It needs to come from the principal to say no*,* this slot they cannot have anything else in it. They should at least t have the opportunity to do sports. Whereas at the moment they are actively being told not to [P15].*

### The need for systemic change

Staff felt the undervalued and peripheral role of physical activity in further education was influenced by the structural approach to its provision across the sector, including the absence of timetabled physical activity for all students. Some participants felt a more systemic change to physical activity provision in further education is needed by making it mandatory. This was contextualised by comparing physical activity provision in FE with approaches in secondary school and in comparable education settings in ‘*other countries’ [P20]* (such as *‘USA’ [P28]*). For them, this highlighted the traditional undervaluing of physical activity in FE in England.

*It’s not compulsory for them to do it (physical activity). The government*,* once they’re plus 16*,* they’re not fussed (about physical activity engagement)*,* which I find terrifying. [P03].*

*I dream of a time where every one of our 4000 learners can select a sport and come play*,* and it’s in their timetables [P14].*

Conversely, several staff cautioned that making physical activity compulsory might be negatively received by students.

*Once you get students who were 16 plus*,* they’re not far off being adults*,* they need to be making their own choices. You start telling them they have to do something. They’re gonna have negative associations with it. [P17]*

However, participants believed the necessary structural shift needed to better embed physical activity within college life and create more opportunities during college hour would have to come from Government policy. Staff felt the lack of prioritisation of or incentivization for physical activity within policy strongly contributed to the feeling that it is treated as an *‘extra’ [P28]* at college, rather than a universal and consistent part of college life.

*SMT (senior management team) is not telling me “you need to get your students to do sport or physical activity”. They’re telling me you need to get students to pass the courses and you need to get them a job. They are telling us that because that’s what OFSTED (Office for Standards in education*,* Children’s Services and Skills) and that’s what the DfE (Department for Education) are telling them. So it’s a top-down thing. [P01]*

Participants also noted the lack of guidance from government on the provision of physical activity within colleges, highlighting that to achieve any significant shifts would require fundamental change to how further education providers are funded and evaluated. Staff suggested that inspection frameworks, such as OFSTED, need to provide clear criteria for physical activity opportunities, facilities and provisions to help achieve a significant cultural shift.

### Theme 3: negative attitudes towards physical activity

#### The impact of negative previous experiences with physical activity

Several participants noted that many students have developed negative attitudes towards physical activity, which negatively impacts their engagement at college. Participants felt these attitudes stem from previous unwelcome experiences, mostly from young people’s encounters with physical education (PE) in secondary school or sports clubs, which leave a lasting impression:

*Many of them have come from schools with a really bad experience of PE…when they get to college age*,* they’re told I don’t have to do PE anymore “Brilliant”.* [P14]

### Aversion to competitive and traditional sport

Participants noted many students have negative attitudes towards sport, particularly competitive environments. This issue was compounded as staff noted that currently, the dominant opportunities for activity within colleges are traditional, competitive sports like football, rugby, hockey or cricket:

*Maybe they aren’t into sport*,* and if not then there’s not really anything for them provided in the college. [P26]*

These data suggest that a reframing of physical activity as not synonymous with sport could support engagement among students. Staff felt that by promoting physical activity in a different light may help counter negative attitudes. Participants recommended greater emphasis on *‘enjoyment’ [P24*], *‘wellbeing’ [P08]* and inclusivity rather than competition, ability or achieving targets or grades.

Some colleges did offer physical activities designed to be more fun, social and non-competitive, such as having a social and participatory pathway for sports for those who wanted a more casual and fun experience as part of their enrichment programme, compared to the talent and performance pathway for students seeking competitive options. However, for most colleges, staff mentioned the focus remains on sports and lacks variety. This does not deter these students’ fears:

I think when people hear the word sport, there’s a feeling of dread. [P02]

Suggestions for alternative physical activity to incorporate a wider range of students’ interests included yoga, running, water sports, gardening, walking groups and skateboarding. Participants also suggested moving away from a commitment model to something more flexible: rather than asking students to choose a sport or activity that they need to stick with, give students a licence to “dip in and out of” different activities and try new things as they move through college. College staff themselves primarily spoke about sport when asked about activity, suggesting that shifts in attitudes and assumptions are required to reframe physical activity to align with less competitive and more incidental and everyday movement activities alongside the formal sport provisions. Participants also described how a strong fear of failure amongst many students means they would rather avoid engaging in competitive environments at all to minimise the risk of failing on front of others and experiencing that embarrassment.

### Gendered barriers to physical activity engagement

Participants discussed how these issues affecting students’ attitudes towards and engagement with physical activity can differ between subgroups of students. Staff emphasised this point for female students in particular, mentioning they felt female students typically engage in less activity at college than male students due to lack of interest and possibly due to a perceived gap in ability and competency compared to boys.

*I think the girls generally are probably more reticent about getting involved in physical activity… I’m being massively generalizing*,*.girls just simply won’t do it. [P20]*

This sentiment may indicate that college staff hold assumptions and stereotypes about female students’ physical activity engagement and competence, which could influence the opportunities offered and discourage participation.

There was also a perception that girls were put off engaging in physical activity at college due to a fear of being sexualised and body shaming/image concerns. One staff member mentioned there had been a very well-attended female sports team, but many stopped showing up after boys would gather outside the hall and watch:

*Our netball team was quite strong and the college encouraged people to come in and watch*,* but many stopped playing because some lads came along watching the girls. [P15]*

Another staff member mentioned the influence of sports gear and kit on females’ fear of sexualisation and body shaming (particularly in mixed gender environments), highlighting this as a key barrier to engagement:

*“Girls are just going to go*,* ‘I’m gonna look fat and ugly in that dress. I’m definitely not going to do that sport’. [P03]*

Staff suggested that colleges could provide female-only physical activity options in the future, but this would need to be explored with students if this would improve physical activity for young women at college:

#### Theme 4: practical challenges to physical activity provision

##### Financial and funding constraints

As well as cultural and structural issues, practical challenges to improving the use of physical activity to support mental health emerged. Financial limitations were cited as a key barrier to the provision of more physical activity opportunities for students within colleges: *“it’s just money. Its dead simple. It simply comes down to the money” [P21]*. Colleges need to pay for the facilities, their maintenance and equipment, along with staff to supervise and facilitate activities. However, staff noted that physical activity opportunities are not directly funded. Instead, colleges receive funding per student, which is intended to cover the entire educational experience: teaching, staff, facilities, learning materials and resources. It falls to the college to decide how to distribute this budget, and what areas to prioritise:

*If you’re saying we’re spending X amount on sport and physical activity*,* yet we haven’t got enough laptops or books or seating or a roof needs fixing then where do we put our resources? [P24]*

Top-down changes to how colleges are funded and how physical activity is prioritised within these structures was a key issue to address according to staff:

*It’s getting away from the funding for obvious success. They’re an easy way of measuring…There’s got to be another way of gaining funding. [P15]*

Participants also reflected on how students’ own personal financial barriers impacted physical activity engagement. Physical activity opportunities within college can be very expensive, which is a significant deterrent for college students and their families:

*So spending like £200 pound per term on sport and then you gotta get there and whatever else. it’s not cheap… the cost element is becoming a big problem. [P01]*

Staff described the inequality in access between students from lower socioeconomic backgrounds and those from more affluent families:

*The ones who have more affluent families tend to do a much more varied range of sports*,* so that I feel like they’ve obviously been given the opportunity to try lots of different sports*,* whereas a lot of the students from the other end of the scale may be focused just on one sport or none. [P02].*

This was seen as a particularly relevant challenge for both the rural and urban-based colleges, as they have a high proportion of students from more disadvantaged backgrounds. Staff felt many of these students were significantly limited in their access to physical activity options outside of college, and it is important for colleges to ensure they are providing these opportunities to address these inequalities:

*As a young person or with the adults that we are working with*,* who often are in receipt of benefits. It’s vital we provide those students with opportunities that they wouldn’t normally have. [P25].*

#### Facilities and inequalities in infrastructure

Even with increased financial backing to offer these opportunities, colleges are also limited to what they can provide, in terms of physical activity, based on the infrastructure they have in place. Crucially, even when colleges were *‘blessed’ [P21]* to have facilities for activities, such as sports grounds and indoor halls, due to increasing demand for teaching space amid growing numbers of students, they were having to use these sports spaces for teaching.

A key difference in infrastructure was identified between rural and urban contexts. While the rural-based colleges tended to have more space and facilities to provide physical activity, urban colleges, had a lack of space, particularly green spaces:

*But at our city centre campus*,* there’s really nothing and we’re right in the middle of a city. There’s a real lack of green spaces nearby. There’s encouragement for students to get out and get moving because there’s no space for that. [P17]*

This lack of space meant they were forced to rely on external partnerships to facilitate any activities, which seemed to result in a lack of physical activity provision and opportunity, as it compounded the funding problem with the need to pay for such activities. Conversely, students in urban contexts have more potential for active commuting, such as walking or cycling in comparison to their counterparts in rural contexts who are more reliant on car travel due to poor transport links. This suggests that for rural colleges in particular, a focus on physical activity opportunities on campus is essential. In both urban and rural contexts, participants pointed to the need for more resources in terms of facilities and more staff to deliver activity opportunities. Several staff recommended a more joined-up approach between education providers and local community organisations, like sports clubs, to share resources and provide further opportunities for their young people.

## Discussion

This study explored further education staff’s perspectives on the role of physical activity in supporting student mental health, identifying a number of cultural, structural and practical barriers as well as opportunities for the future. Consistent with national evidence showing rising levels of poor mental health amongst young people [18], participants described mental health as one of the most pressing challenges facing FE settings. Staff viewed physical activity as a promising but currently underused strategy to address this. The findings extend prior research from schools and higher education settings, by detailing how FE colleges conceptualise physical activity in relation to mental health and opportunities for the future as a strategy to support student mental health.

A key finding from this study is that mental health and physical activity are not generally connected within the FE sector. This fits with previous work showing there are very few interventions that connect mental health and physical activity within education settings at the FE level [9]. At a student level, this aligns with previous evidence that young people often have limited awareness or recognition of the psychological benefits of physical activity, despite strong empirical support for its efficacy in improving mental health outcomes such as anxiety, depression and stress [7, 19–21]. For example, previous research in UK higher education settings has suggested that while young people recognise that physical activity and mental health are connected, they view these as distinct and separate elements. Physical activity is predominantly considered a tool for physical health [15, 21]. Our findings extend understanding to the views of FE students; rather than solely due to a lack of awareness of the connection, staff believe students do not view the potential benefits of PA for mental health as relevant to them or have a lack of belief in the effects [15, 21, 22]. One future strategy for FE colleges is to better connect physical activity to meaningful personal reasons, and to more explicitly tie it to mental health and wellbeing. Promotional or educational initiatives during the first few weeks and throughout a student’s time at college could explicitly frame physical activity as a strategy to achieve mental health and wellbeing [23, 24].

This missing link between physical activity and mental health was underscored by a wider structural issue identified around physical activity not being universally considered (and promoted) as a part of what FE colleges deliver. Social norms and values within the education environment can be an important determinant of student behaviours and are increasingly recognised as holding important influence over the physical and mental health and wellbeing of young people [25]. This ‘culture’ can be shaped by various factors including school leadership and teachers, the physical environment and the goals and priorities of the institution [25, 26]. The perceived value or importance that school leaders and the institution place on physical activity is a key factor influencing student engagement; the more students perceive that physical activity is valued within their educational institution, the higher the likelihood of increased participation and engagement [15, 27, 28]. Our participants’ accounts of the peripheral positioning of physical activity as an optional add-on only for those interested in sport, seemed to stem from a widespread lack of prioritisation of physical activity across the FE sector. Staff saw a culture shift in FE as vitally important to promoting physical activity, although no participants mentioned role modelling or integrating physical activity into teaching activities, which we interpreted as symptomatic of the pressure within colleges to focus on good grades (as a financial and OFSTED imperative) that impacts on teachers’ capacity for physical activity promotion as well as the students. The importance of physical activity across college life could be reinforced in the future through staff training, better preparing staff to promote and model physical activity as part of the everyday culture within FE settings.

Participants felt that physical activity engagement among FE students was particularly vulnerable to de-prioritisation and competition from academic and other demands, as it is neither required nor embedded within curricula, as it is in secondary school. Previous evidence suggests stronger frameworks and requirements for physical activity within education policy is linked with higher levels of engagement [29]. Recent work has suggested that 80% of adolescents report the education setting as their only source of physical activity, which may be partly explained by schools being a primary setting for adolescents’ activity due to the mandatory nature of PE or physical activity [30]. This is particularly relevant in light of our findings that FE students face considerable constraints on their time outside of college through part-time jobs, substantial commuting time and other responsibilities, making it important that future initiatives are not placing additional requirements on student’s time and inadvertently causing more stress. Although staff held differing views on whether physical activity should be mandatory in the FE setting, a commonly suggested compromise emerged which involved the FE sector moving towards a mandatory approach for physical activity but with considerable choice built in. In this model, all students would have timetabled, protected opportunities for physical activity, but students can choose what they do based on their interests and capabilities This would align with the finding that autonomy-supportive approaches increase engagement in adolescents [31].

Although a mandatory approach in this way may help to increase participation, it may also provoke resistance amongst college students, particularly given our findings around negative attitudes towards physical activity being a major barrier to engagement. These findings align with previous studies that adolescents commonly disengage from physical activity due to fear of judgement, performance anxiety or negative experiences within school sport and PE [15, 32]. These issues may be particularly relevant within FE, where young people suddenly experience a lot more independence and autonomy, and options for engaging in physical activity are often limited to competitive and sports-based activities. Widening the scope of physical activity provision in FE is needed, including delivering activities like walking, dance, yoga, or non-sport exercise like gym access, cycling or running, along with alternative activities like skateboarding or gardening; much of these can be acceptable and effective for supporting the mental health of young people when delivered within education settings [9, 15]. While diversifying physical activity options within FE may be a big step towards increasing student engagement there is also a change needed to how it is promoted and encouraged. Our findings point to an opportunity for colleges to not only reframe physical activity to better connect it with mental health, but to also emphasise fun, enjoyment and participation rather than competition or performance. This aligns with evidence supporting a student-centred approach to physical activity provision, prioritising elements like autonomy, socialising and fun as facilitators to physical activity engagement [15, 22, 32].

Further focus may be necessary to address female students’ specific barriers mentioned in this study. Mirroring previous evidence regarding a gender disparity within young people’s physical activity engagement, female college students were perceived to be less active than their male counterparts [33]. An important step to improving this situation is to address the gender-specific barriers mentioned such as body image concerns, fears of observation and sexualisation and perceptions of being less competent; these are also well documented issues in the literature [15, 32]. Female-only physical activity options was one suggestion, and despite mixed results, some prior evidence suggests this may increase female student’s acceptability and participation in physical activity [34, 35]. One previous initiative within FE colleges reported positive impacts of female-only options [36]. This program also reported beneficial effects from strategies like conducting sessions in more private, unobservable spaces or glow sports under UV light where students can’t be seen [36]. While these strategies may be valuable in addressing such issues, we argue these should be positioned as part of a broader, more inclusive approach to avoid reinforcing gendered stereotypes about female physical activity engagement. Alongside expanding the range of options available, including non-competitive activities, strategies to actively promote female participation should be implemented, such as focused encouragement and support for girls to take part in physical activities, flexibility around clothing to support comfort and acceptability and fostering environments that minimise judgement and performance pressures [15, 37–39]. Such actions could help increase female student physical activity engagement without fostering segregation and perpetuating stereotypes which can further discourage engagement.

However, increasing physical activity provision through such strategies would require more resources and funding along with appropriate infrastructure, which are significant challenges for FE institutions. Our findings suggest the need for systemic change to funding structures, away from solely prioritising academic outcomes. However, even with funding shifts, colleges in urban contexts with major competition for space may need additional strategies that funding itself cannot overcome. Strategies such as better partnerships between colleges and local clubs, leisure centres, or sports organisations have been supported previously, and may help to reduce resource burden on colleges and expand access to physical activity opportunities and facilities for young people [12, 40, 41].

This expansion of the provision of and access to physical activity opportunities within college was seen as vital, due to the financial barriers identified for college students. Colleges typically cater for a high proportion of students from socioeconomically disadvantaged backgrounds, and participants discussed how many students face difficulty in affording physical activity opportunities, sports club fees, equipment and appropriate gear [12, 13]. This aligns with the evidence of the association between socioeconomic status and physical activity engagement, where those from more affluent backgrounds report higher levels of activity and greater access to organised sport opportunities [42, 43]. Given that further education settings have a high proportion of students from lower socioeconomic backgrounds, colleges may represent an important context for addressing these inequalities by providing low-cost, inclusive and accessible opportunities for physical activity within the college environment. Without appropriate investment and enhanced provision, existing disparities in physical activity engagement risk being reinforced or exacerbated, with potential downstream consequences for both physical and mental health.

Our participants emphasised that even with this need to increase funding to widen physical activity provision in FE to better support student mental health, they believe such investment should be viewed as a cost saving initiative in the long-term. This aligns with wider public health evidence suggesting the substantial positive return on investment of prevention interventions and long-term economic benefits; particularly for school-based mental health prevention programmes for adolescents [44]. This would require significant shifts in how policy-makers conceptualise funding for physical activity and mental health provision in the FE sector, needing top-down prioritisation and dedicated funding to ensure physical activity is seen as a core component of a student mental health support strategy within colleges. We have outlined several significant implications based on our findings for the future in Table 3, 4.

**Table 4:** Staff-suggested opportunities and recommended actions for the future

Theme	Description	Recommended action	Action examples
“A missing link” between physical activity and mental health	Staff identified a clear disconnect between physical activity and mental health: students did not typically recognise physical activity as beneficial for their mental health, and FE colleges tended to address physical activity and mental health separately in their promotion, provision, and within support services.	**Institutional action**: Implement educational and promotional activities that make the connection between physical activity and mental health explicit, increasing students’ understanding and valuing of its mental health benefits.	• Integrate recurring tutorial‑based sessions that teach students about the mental‑health benefits of physical activity. • Include a physical activity and mental health promotional component during new student’s induction that introduces key messages about physical activity’s mental‑health benefits and explains how students can access activities throughout the year.
		**Institutional action**: Need for a joined-up approach to physical activity and mental health within colleges, and to formally integrate physical activity within student support services.	• Integrate physical activity within student support services as part of routine student wellbeing provision. • Establish formal referral pathways so that students seeking mental health support can be offered access to physical activity opportunities delivered by PA or sport teams, if appropriate. • Strengthen collaboration between physical activity staff and student support/wellbeing teams through shared responsibilities, joint working protocols, and coordinated support plans.
A perception that physical activity is not a core part of Further Education	Staff perceived physical activity is not a core part of FE, rather it occupies a marginal place as an optional add‑on for sports-interested students. As a result, physical activity is deprioritised within college structures, limiting its engagement and accessibility for the wider student population.	**Institutional action**: Colleges need to more actively promote and prioritise physical activity as a core, accessible and valued part of Further Education for all students.	• Implement more consistent and widespread promotion of physical activity opportunities and specific steps on how to access them across the college, including through student emails, newsletters, and visible on‑site promotional materials • Senior college leaders need to ensure timetabled enrichment periods are better protected by preventing extra academic lessons, revision classes, or additional work from being placed in these slots, enabling equitable access for all students. • Introduce protected physical activity slots within the timetable, for example, a 30‑minute daily period for students to be active in line with recommended guidelines. • Include modelling and promotion of physical activity within staff training to reinforce the importance of physical activity across college and implement within the college day for students.
		**Policy action**: Specific and consistent sector‑level policy guidance is needed to ensure physical activity is a core, valued component of FE provision	• DfE to establish a national framework for physical activity guidance in FE, including explicit criteria and expectations for physical activity provision in colleges. • DfE to explore a sector‑wide model for protected, timetabled physical activity in FE. • Introduce clearer Ofsted criteria within inspection frameworks evaluating colleges’ physical activity provision to incentivise consistent prioritisation of physical activity.
Negative attitudes towards physical activity	Staff described negative attitudes towards physical activity among many students which hampered engagement, shaped by past negative experiences, fear of failure or judgement from others and discomfort with competitive environments. Staff felt these barriers were exacerbated by the college’s predominately competitive‑physical activity offer.	**Institutional action**: Future provision should be designed to increase physical activity engagement for all students by recognising and addressing student‑level barriers.	• Expand the scope of physical activity opportunities at college beyond traditional sport and competitive options. • College leaders, teachers & physical activity teams should collaborate with students to provide non-sport, alternative or informal activities, matching student interests and preferences. • Focus promotion of physical activity to emphasise fun, social connection, enjoyment, and wellbeing rather than competition or performance. • Collaborate directly with specific student groups (e.g., female students) to identify and address their unique barriers to engagement, and co‑design physical activity opportunities that reflect their preferences and needs.
Practical challenges to physical activity and mental health provision	Staff identified a range of practical challenges that limited physical activity and mental health provision, including financial and funding constraints, limited infrastructure, and unequal availability of suitable physical activity spaces across colleges	**Institutional action**: Build partnerships with community organisations to increase access to physical activity opportunities and reduce resource burden.	• Develop partnerships and agreements with local sports clubs, coaches and facilities. Colleges could refer students to clubs for physical activity opportunities, and clubs could facilitate access to resources, expertise and infrastructure. • Build partnerships with local gyms to offer discounted student membership prices or facility usage.
		**Policy action**: Changes to how colleges are funded to provide direct support for physical activity and mental health provision.	• Implement a specific funding stream for physical activity and mental health provision, allowing colleges to invest in dedicated staff roles, accessible activity spaces, and ongoing programme delivery and reducing reliance on overstretched general budgets. • Incorporate physical activity and/or mental health and wellbeing outcomes within funding and performance frameworks so that colleges are assessed and resourced mot only on academic metrics, but on more holistic student success measures.

While this study focused on FE settings, the perceived disconnect between physical activity and mental health described by our participants reflects a broader cultural separation seen across other populations and contexts, which has been flagged as a key issue and research gap [24]. These findings therefore contribute to a wider evidence gap by illustrating how this separation is experienced within FE and by highlighting the cultural and organisational factors that shape the extent to which physical activity is connected to mental health. To address this cultural issue, we need to move beyond interventions that support behaviour change, although these can be important, to consider how to create organisational and societal change [45]. This may include national policy that mandates provision of universal physical activity provision in FE colleges, as discussed above, as well as considering this for other settings such as workplaces. It also links to increasing interest in the need to design neighbourhoods that incorporate safe and inclusive spaces for everyone to engage in greater physical activity, as a way of improving mental health [46].

### Strengths and limitations

A strength of this study is its detailed, sector-specific exploration of FE staff views, a group rarely represented in young people’s physical activity or mental health research. A qualitative approach across multiple college contexts provided rich insights reflecting differences across the sector, and applying the Framework Method supported analytic rigour. However, there are several limitations to this work, including that the data only represents the views and perspectives of college staff; students’ own perspectives may differ particularly with some of the more nuanced themes like attitudes and beliefs. As the sample comprised staff from five FE colleges within one region, the findings may have limited transferability to other FE contexts. The sample may also be limited by self-selection bias, whereby participants may have signed up to take part as they have particular beliefs or previous experiences, which may reflect more positive attitudes towards physical activity or mental health. We attempted to counteract this through purposive sampling and snowball recruitment of referred staff from higher managerial roles as the study progressed; and a key strength of this study is the recruitment of staff from senior leadership positions who are typically hard to reach.

## Conclusion

The findings of this study suggest that while FE staff view physical activity as a promising tool for supporting the significant challenge colleges face with student mental health, multiple structural and perceptual barriers currently limit physical activity engagement. Participants proposed addressing these challenges will require coordinated action at institutional and policy levels, including the reframing of physical activity within the sector, better integration of physical activity and mental health, diversifying physical activity provision and reforming investment. Future research should explore students’ beliefs and perspectives regarding physical activity as a mental health support option in FE, as well as testing integrated physical activity and mental health models within colleges. Future work could also examine how policy-level reforms might enable scalable and equitable provision of physical activity to support mental health across the sector. Collectively, these findings contribute novel insights into how FE staff understand the relationship between physical activity and student mental health, and highlight the institutional and policy changes needed to position physical activity as a potentially valuable part of college student mental health promotion.

The findings suggest college staff believe physical activity may be a promising avenue of exploration for supporting the mental health and wellbeing of further education students in the future.

## Supplementary Information

Below is the link to the electronic supplementary material.

Supplementary Material 1.

## Data Availability

The underpinning research data will be published as an anonymised dataset at the University of Bristol Research Data Repository (data.bris.ac.uk/data) at the end of the lead researcher’s PhD studentship. It will be published as a restricted access dataset; only accessible to bona fide researchers. This level of access is required for ethical reasons and to protect the anonymity of participants. All participants confirmed their permission for the data to be published as an anonymous dataset at the University of Bristol Research Data Repository at the time the data was generated. Once published, a metadata record will be published openly by the repository and this record will clearly state how data can be accessed by bona fide researchers. Requests for access will be directed to the Research Data team at Bristol, who will assess the motives of potential data re-users before granting access to the data. No authentic request for access will be refused and re-users will not be charged for any part of this process.
