# Supplementary material for: A qualitative exploration of further education college staff perspectives on the challenges and opportunities for using physical activity to support student mental health in England
Source: Discov Public Health. 2026 Sep 6;23(1):1426. doi: 10.1186/s12982-026-02817-x (PMC13547178; doi:10.1186/s12982-026-02817-x)
Supplement: Supplementary file 1 [file 12982_2026_2817_MOESM1_ESM.docx]

**College staff member interview topic guide**

**Identifying strategies to support and improve the mental health of young people attending further education through physical activity**

**Introduction**

- Introduce self, thank staff member for their participation and time and introduce the research and explain briefly what the interview will be about:

*This research project aims to identify strategies to support the mental health of students in further education through physical activity. Physical activity could include organized exercise sessions such as team sports, going to the gym or lifestyle activities such as walking or cycling. This study is part of a project that will also include further research with students and young people, and aims to inform the development of future recommendations of physical activity-based interventions to support students’ mental health.*

*As part of this, I am interested in speaking to teachers and staff members who work closely with students in the college setting, or employers who work with Further Education students in apprenticeships, to get their input and views on this topic. Today, I am interested in talking to you about how your college promotes and encourages physical activity and mental health, what helps or hinders these types of initiatives and your insight into opportunities that may exist to improve student mental health through physical activity in the future.*

- Confirm they have read information sheet and understand they can leave at any point during the interview.
- Reconfirm consent on audio recording and ensure recorder is switched on

| **Q1** | **Ice breaker / Warm Up** | **Prompts** |
| --- | --- | --- |
| What is your position in the college, can you tell me about your role? | | - *How long have you been in the role?* - *What kinds of students do you engage with in your role?* |
| When it comes to mental health and physical activity, in your role, is there one of these topics you are more interested in/have more engagement with? | | - *would you have more of an insight/expertise/involvement with physical activity or mental health?* *Or is it both?* |

| **Q2** | **Physical Activity** | **Prompts:** |
| --- | --- | --- |
| Q2(a) | Can I firstly ask you a little bit about the physical activity of the students, what kinds of physical activity do students at the college typically engage in? | - *Do you think students are getting sufficient physical activity each week?* - *Do you think some groups are more or less active than others?* - *How does the college context impact student physical activity? Do you think the (Urban/Rural) setting has an impact?* - *How do students typically commute to college?* - *Do students have physical education as part of their course? What does this involve?* |
| Q2(b) | **Promotion of student PA:** how does the college encourage or promote physical activity among students? | - *Are there any college initiatives in place to promote physical activity among students?* - *What physical activities are provided/encouraged among students?* - *What facilities does the college have for physical activity, such as gyms, pitches, equipment etc?* |
| Q2(c) | **(if there are no PA initiatives)** why doesn’t the college have initiatives to promote physical activity? | - *What are the barriers, what is stopping the college from implementing these kinds of initiatives?* - *Do you know if the college had initiatives to promote PA in the past? Why do you think they were stopped?* |
| Q2(d) | **(If there are PA initiatives)** Do you think they are successful in promoting physical activity?  What helps these initiatives in promoting PA among students? | - *How do students feel about these initiatives? Has there been feedback?* - *Are they being used? (encourage them to think about different student groups here, such as students on different courses, genders, SES etc).* |
| Q2(f) | In your opinion, **what opportunities might exist to promote physical activity among students at the college?** | - *What changes would you make in the promotion of physical activity at the college?* - *How would you recommend to implement a physical activity initiative? What resources would this need?* - *Do you think there are any opportunities to integrate physical activity into the college day/week/year?* - *What would you do to increase uptake of physical activity among students? How would you increase uptake of physical activity among certain students?* |

| **Q3** | **Mental Health** | **Prompts:** |
| --- | --- | --- |
| Q3(a) | I would like to ask you about the mental health of students, **what are the main challenges to the mental health of young people** attending the college? | - *How are these challenges the same or different to all young people of a similar age?* - *Do students at this college have any particular challenges to their mental health that may be different to other colleges/location? (e.g. to do with levels of SEN or deprivation, rural/urban nature)* - *Do you notice any changes in student mental health over the course of the academic year/seasonal changes?* - *How do students manage their own mental health?* |
| Q3(b) | What does the **college currently do to support the mental health of students**? | - *Are there any college initiatives in place to promote mental health among students?* - *What services are available to a student at the college to support their mental health if they have an issue?* - *How are these communicated to students?* - *How do students feel about these services? Has there been feedback? Are they used?* - *What does the college feel is their responsibility to the wellbeing of students?* - *How do these services apply to different groups of students, such as part-time students or those on apprenticeships/internships?* |

| **Q4** | **Physical Activity & Mental Health** | **Prompts:** |
| --- | --- | --- |
| Q4(a) | Do you seen any **connections between the physical activity and the mental health** of students? | - *Do the college staff think about these things together?* - *Are similar people responsible for both? Why hasn’t these two been connected in terms of student wellbeing?* - *How do you think physical activity impacts student mental health? How do you think mental health impacts physical activity?* |
| Q4(b) | Thinking about how the college could promote physical activity to support the mental health of students, how would you recommend implementing these kind of initiatives? | - *Are there any kinds of activities you would suggest would be impactful for students’ mental health?* - *Who/when/how do you think would be best to deliver these kinds of initiatives? Would there be barriers to implementing these kinds of initiatives in the future? What would help implementing these in the future?* - *Do you think increasing PA among students would be helpful for supporting their mental health?* |
